# Characterizing Distributions of Tensile Strength and Crack Precursor Size to Evaluate Filler Dispersion Effects and Reliability of Rubber

**DOI:** 10.3390/polym12010203

**Published:** 2020-01-13

**Authors:** Christopher G. Robertson, Lewis B. Tunnicliffe, Lawrence Maciag, Mark A. Bauman, Kurt Miller, Charles R. Herd, William V. Mars

**Affiliations:** 1 Endurica LLC, Findlay, OH 45840, USA; mabauman@endurica.com (M.A.B.); wvmars@endurica.com (W.V.M.); 2 Birla Carbon, Marietta, GA 30062, USA; Charles.Herd@adityabirla.com; 3 Axel Products, Ann Arbor, MI 48104, USA; larry@axelproducts.com (L.M.); kurt@axelproducts.com (K.M.)

**Keywords:** tensile strength, Weibull statistics, rubber durability, crack precursors, filler dispersion

## Abstract

Undispersed filler agglomerates or other substantial inclusions/contaminants in rubber can act as large crack precursors that reduce the strength and fatigue lifetime of the material. To demonstrate this, we use tensile strength (stress at break, σ_b_) data from 50 specimens to characterize the failure distribution behavior of carbon black (CB) reinforced styrene-butadiene rubber (SBR) compounds. Poor mixing was simulated by adding a portion of the CB late in the mixing process, and glass beads (microspheres) with 517 μm average diameter were introduced during milling to reproduce the effects of large inclusions. The σ_b_ distribution was well described with a simple unimodal Weibull distribution for the control compound, but the tensile strengths of the poor CB dispersion material and the compounds with the glass beads required bimodal Weibull distributions. For the material with the lowest level of glass beads—corresponding to less than one microsphere per test specimen—the bimodal failure distribution spanned a very large range of σ_b_ from 13.7 to 22.7 MPa in contrast to the relatively narrow σ_b_ distribution for the control from 18.4 to 23.8 MPa. Crack precursor size (*c*_0_) distributions were also inferred from the data, and the glass beads introduced *c*_0_ values in the 400 μm range compared to about 180 μm for the control. In contrast to σ_b_, critical tearing energy (tear strength) was unaffected by the presence of the CB agglomerates and glass beads, because the strain energy focuses on the pre-cut macroscopic crack in the sample during tear testing rather than on the microscopic crack precursors within the rubber. The glass beads were not detected by conventional filler dispersion measurements using interferometric microscopy, indicating that tensile strength distribution characterization is an important complementary approach for identifying the presence of minor amounts of large inclusions in rubber.

## 1. Introduction

Rubber fracture mechanics can be used to predict fatigue lifetime of an elastomer product, and the essential inputs are the crack growth rate law and the size of crack precursors [1,2,3]. Our research investigation focuses on the microscopic crack precursors—also referred to as intrinsic flaws or defects—and how to effectively characterize their size distribution. Crack precursors can arise from undispersed filler agglomerates, regions of high crosslink density from incomplete dispersion of curatives, hard contaminants within the raw materials (e.g., dirt in natural rubber or grit in carbon black) or introduced during the processing of the rubber, and bubbles/voids within the material. Due to their heterogenous origins, crack precursors are not uniform in size or shape, and the distribution of crack precursor sizes in a rubber material leads to a distribution of failure properties, such as fatigue lifetime and tensile strength.

Direct observation of crack precursors was documented with microscopy by Huneau et al. [4]. Carbon black filled natural rubber was fatigued, and then scanning electron microcopy (SEM) with energy-dispersive X-ray (EDX) elemental analysis was used to identify the chemical make-up of the sources for the noted micro-cracks. The majority of cracks were initiated from zinc oxide particles and carbon black agglomerates. We comment, however, that the images of presumed carbon black agglomerates in Figures 9 and 11 of that publication actually depict particles of ball coke, which is an impurity that can be produced in CB manufacturing [5].

While particle-related influences like undispersed filler agglomerates can certainly be the source of larger crack starters in rubber compounds, even unfilled elastomers with simple cure systems have precursors that can be quantified from fatigue testing and strength measurements. Choi and Roland [6] reported *c*_0_ in the range from 10 to 29 μm for various types of natural rubber that were formulated only with an antioxidant and a peroxide for crosslinking the materials.

The statistical occurrence of precursors/defects within the actively deformed volume of a test specimen or real rubber product is an important aspect of durability. For the same rubber compound, the fatigue lifetime decreased as the test sample volume was increased—larger cylindrical dumbbell samples failed at lower number of loading cycles—in a study by Ludwig et al. [7]. The corresponding precursor sizes for the three dumbbell shapes investigated were determined to be 100, 125, and 150 μm in order of increasing specimen test volume, reflecting the greater chance of bigger precursors occurring in larger sample volumes. The crack precursor size and its distribution are critical to elastomer reliability, but number density considerations are also important.

Ignatz-Hoover and coworkers [8,9,10] developed an effective way of quantifying raw material effects and processing influences related to the dispersion of rubber ingredients including insoluble sulfur, vulcanization accelerators, and silica filler in various rubber compounds. Tensile strength of rubber is sensitive to the size of crack precursors, with larger precursors from poorly dispersed raw materials leading to lower strengths. They used Weibull statistics to fit the tensile strength (stress at break, σ_b_) data from testing of numerous replicate rubber specimens, with 50 specimens determined to be an efficient population size for characterizing the failure distribution to give quantitative insights into dispersion effects [10].

Our research further investigates the utility of tensile testing of 50 specimens to quantify the σ_b_ distribution in rubber, and we expand the approach to include assessment of the related crack precursor size (*c*_0_) distribution. This effective characterization of *c*_0_ distribution is key to fatigue lifetime modeling efforts to predict the reliability of rubber products, as will be discussed. The rubber compounds studied are based on carbon black (CB) filled styrene-butadiene rubber (SBR), with intentional poor mixing of CB and addition of glass microspheres used to emphasize the influence of inclusions on tensile strength and reliability of rubber. We compare the resulting crack precursor size distributions with defect size distributions measured using microscopy to illustrate that assessing *c*_0_ distribution from tensile strength testing is an important complementary approach for identifying the presence of minor amounts of large inclusions in rubber that cannot be detected by conventional filler dispersion measurements.

## 2. Experimental Details

### 2.1. Materials, Mixing, and Curing

**Figure 1:**
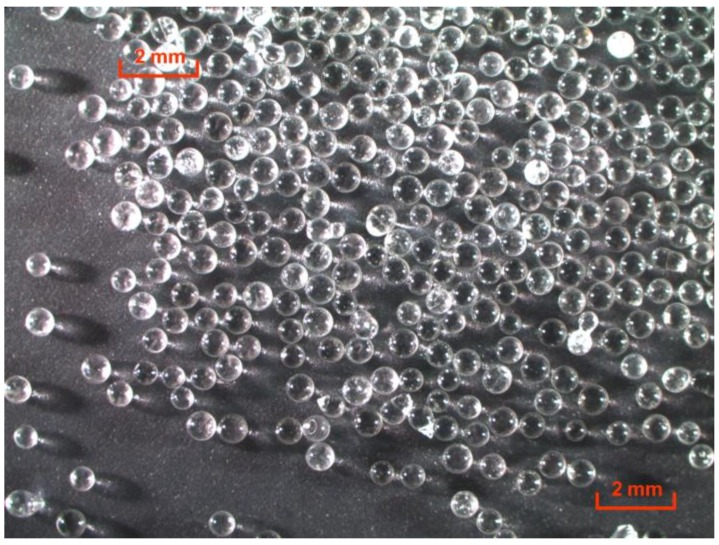
Optical microscope image of glass beads used in this study.

Rubber compounds were mixed using a Farrell Banbury 1.5 L internal mixer (Ansonia, CT, USA), and the formulations in units of parts per hundred rubber (phr) are shown in Table 1. The master batch (non-productive stage) involved mixing for 5 min with mixer cooling water temperature of 40 °C using a rotor speed of 77 rpm, which was adjusted as needed during the last two minutes of mixing to reach the drop temperature of 150 °C. The final batch (productive stage) involved mixing for 4 min with mixer cooling water temperature of 25 °C using a rotor speed of 60 rpm, which was adjusted as needed during the last two minutes of mixing to reach the drop temperature of 100 °C. The carbon black material used is an ultra-clean N550 grade having 0 ppm particulate residue recoverable after passing through 325 mesh screen [11]. A fine (active) grade of zinc oxide, ZnO 35, was used which has a surface area of 45 m^2^/g, which is very similar to the N550 CB surface area of 40 m^2^/g. A compound with poor carbon black dispersion (Poor Disp.) was created by adding 40% (20 phr) of the carbon black one minute before the end of the productive mixing stage. Solid glass beads (microspheres) were purchased from Sigma-Aldrich (product ID G8772; St. Louis, MO, USA) and have a density of 2.5 g/cm^3^ as reported by the manufacturer. Size measurements of the microspheres were conducted using a Horiba LA 960 laser diffraction particle size analyzer (Piscataway, NJ, USA), which yielded diameters in the range from 394 to 678 μm for 98% of the population, with a most probable diameter (d) of 517 μm (0.517 mm). The microspheres are shown in Figure 1. A two-roll mill was used to add the glass beads to two rubber compounds, one at a lower level of 0.09 phr (Bead Low) and the other at a higher level of 0.72 phr (Bead High). The mill gap was 1.5 mm (3 times the bead diameter), and the mill temperature was 70 °C during mixing the beads into the rubber. The four rubber compounds were compression molded and cured for 13 min (~T90 + 5 min) at 160 °C to form 150 mm × 150 mm × 2 mm thick sheets for testing.

### 2.2. Tear Testing

Critical tearing energy testing was conducted on the 2 mm thick sheets of cured rubber using a planar tension (pure shear) geometry with 150 mm width and 10 mm gauge height with a 25 mm initial edge cut at one side. An Instron Model 5866 Electro-Mechanical Test Instrument with a 10 kN load cell (Instron Corp., 2525-804 10 kN; Norwood, MA, USA) was employed for the testing. For accurate strain measurements, a laser extensometer was used (Electronic Instrument Research Model LE-05, Full Scale 125 mm; Irwin, PA, USA). The pre-cut crack was opened by stretching the specimen at a crosshead travel rate of 0.1 mm/s until tearing occurred. The critical tearing energy (*T*_c_) was determined from the strain energy density (*W*, integration of stress-strain curve) at the point of tearing multiplied by the initial specimen height of 10 mm.

### 2.3. Tensile Testing

Test specimens were cut from the 2 mm thick cured rubber sheets in the mill direction using a DIN 53504-S2 dumbbell cutting die, which has a gauge length of 25 mm and a width of 4 mm. The 2 mm thickness is a nominal dimension, and the actual test specimen thicknesses ranged from 2.0 to 2.2 mm with an average of about 2.1 mm. Simple tension testing of each specimen was performed using an Instron Model 5864 Electro-Mechanical Test Instrument with a 1 kN load cell (Instron Corp., 2525-806 1 kN; Norwood, MA, USA). A laser extensometer (Electronic Instrument Research Model LE-15, Full Scale 380 mm; Irwin, PA, USA) was used for accurate strain measurements. Each specimen was extended at a crosshead travel rate of 4 mm/sec until break, and 50 specimens were tested for each compound in order to characterize the failure distribution. Numerical integration of each stress–strain curve was performed in order to quantify the strain energy density at break (*W*_b_), and the stress at break (σ_b_) and strain at break (ε_b_) were also evaluated for each test.

### 2.4. Microscopy and Dispersion Measurements

A Nikon SMZ1000 (Melville, NY, USA) optical microscope was used for imaging the glass microspheres and inspecting the fracture surfaces of the tensile test specimens. Cured rubber specimens of Control, Bead Low, and Bead High materials, with approximate dimensions 40 mm × 40 mm × 2 mm, were scanned using X-ray computed tomography (Zeiss Metrotom 3D CT inspection system; White Plains, NY, USA) to image areas of higher electron density (glass beads and zinc oxide particles). Interferometric Microscopy (IFM) dispersion testing was performed on cut surfaces of the cured rubber sheets using a Zygo New View 5000 microscope (Middlefield, CT, USA). IFM determines quantitative size and distributional values of undispersed carbon black based on 2-dimensional sampling of the interior of the rubber compounds generated from razor cut surfaces. The IFM technique generates a carbon black dispersion index (DI) based upon the measured three dimensional surface roughness of the razor cut rubber sample. The assumption here is that while the razor blade cuts through the pigmented rubber matrix with relative ease, hard defects (such as undispersed CB agglomerates) are pushed back into the body of the rubber sample and subsequently protrude from the cut surface of the rubber compound. Measurement of surface roughness of peaks and valleys can therefore be correlated to CB macro-dispersion state and defect size and number [12,13]. Under standard ASTM D2663 method D setup conditions, the IFM images an area of 825 μm × 825 μm of the rubber compound in a single scan. Ten such scans are run per compound, covering a total area of 6.8 mm^2^, and the results are combined and analyzed to provide final statistics. The DI is calculated from the area fraction of topographical features greater than 5 μm in equivalent spherical diameter, and DI = 100 reflects full dispersion by this definition. Defect size and area density values and statistics can also be extracted from the topology scans.

## 3. Results and Discussion

**Figure 2:**
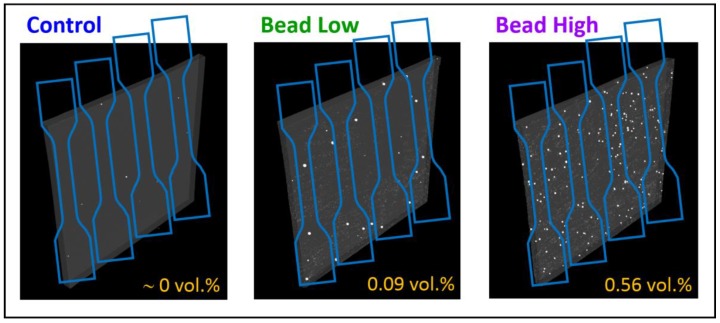
X-ray computed tomography scans for specimens with approximate dimensions of 40 mm × 40 mm × 2 mm. The numbers represent the amount of higher electron density components within each material. Also shown are dimensions of the dumbbell specimens used for tensile testing.

Four rubber compounds were created to study tensile strength distribution effects, and details of the formulations are given in Table 1. We selected an ultra-clean grade of N550 carbon black with 0 ppm residue to avoid any potential influence from filler contaminants. Also, a fine particle grade of zinc oxide was used which has a surface area of 45 m^2^/g, which is very similar to the N550 CB surface area of 40 m^2^/g. The reference compound (Control) was a model CB-reinforced SBR, which was mixed using a standard laboratory mixing protocol. Poor mixing was simulated by adding 40% (20 phr) of the carbon black one minute before the end of the final (productive) mixing stage to produce a compound with poor CB dispersion (Poor Disp.). The final two compounds were generated by mixing the Control compound and then adding glass beads—solid microspheres with average diameter of 517 μm (0.517 mm) shown in Figure 1—at two different concentrations on a two-roll mill after the productive mixing stage. The two levels of microspheres were 0.09 phr (Bead Low) and an eight times greater amount of 0.72 phr (Bead High), corresponding to volume averages of 0.78 bead and 6.24 beads per gauge section region of the specimen geometry used for tensile testing. The X-ray computed tomography (CT) scans presented in Figure 2 confirmed: (1) Two distinct glass bead loadings for Bead Low and Bead High materials; (2) that the glass beads did not fracture during milling into the rubber compounds; and (3) the absence of large zinc oxide inclusions in the Control material.

**Figure 3:**
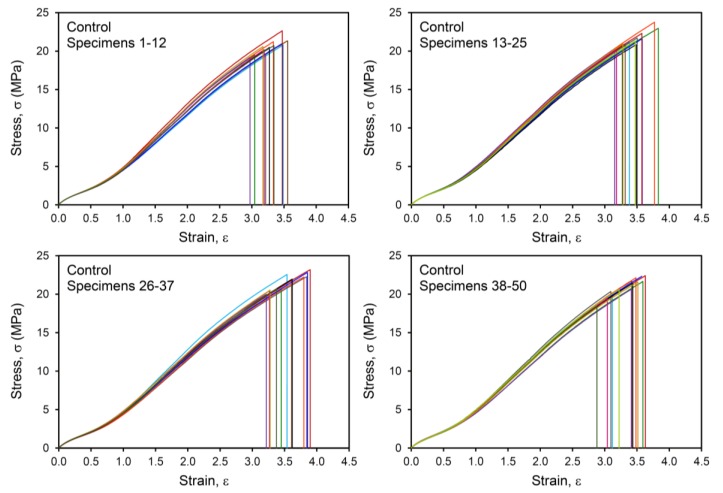
Tensile stress–strain results up to break for Control compound, with data for 50 specimens (replicates) shown using different colored lines.

**Figure 4:**
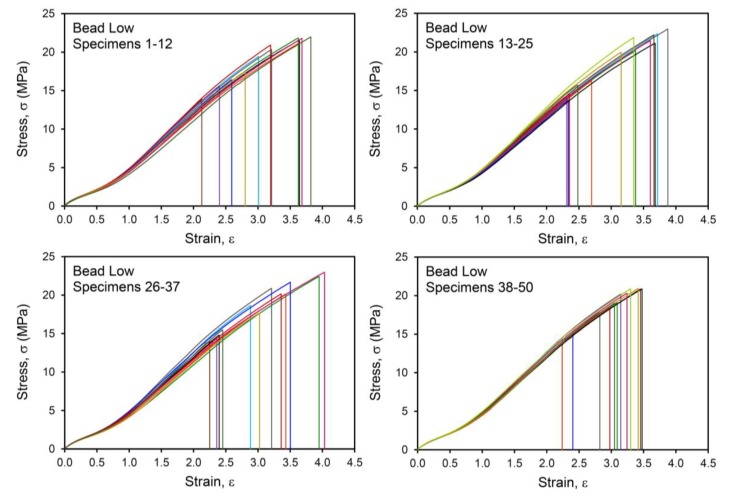
Tensile stress–strain results up to break for Bead Low compound, with data for 50 specimens (replicates) shown using different colored lines.

**Figure 5:**
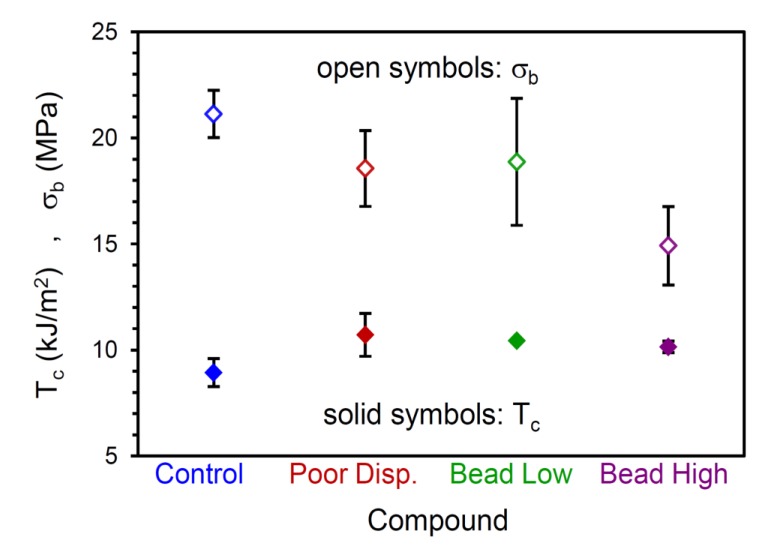
Tensile strength (stress at break, σ_b_) and critical tearing energy (*T*_c_) for the four compounds.

For each material, 50 replicate tensile tests were conducted to characterize the failure populations. Examples of the stress-strain curves are shown in Figure 3 and Figure 4 for the Control and Bead Low materials, where it is immediately clear that there is a broader distribution of failure for the compound with the low concentration of glass microspheres compared to the reference compound. The failure distribution for the Bead Low compound spanned a very large range of σ_b_, from 13.7 to 22.7 MPa, in contrast to the relatively narrow σ_b_ distribution for the Control, from 18.4 to 23.8 MPa. This is reflected in the standard deviations for σ_b_ in Table 2 and Figure 5. It is common to interpret such failure variances as “error bars” coming from combined material and test method variability considerations, but we emphasize that this information is providing insights into real crack precursor size distribution information, as will be shown. The average tensile strength decreased for the Poor Disp. (18.6 MPa), Bead Low (18.9 MPa), and Bead High (14.9 MPa) materials compared to the Control (21.1 MPa), as summarized in Table 2. In contrast to σ_b_, the critical tearing energy (tear strength, *T*_c_) was not affected by poor CB dispersion or the addition of the hard microspheres (Table 2 and Figure 5). In a tear test, the strain energy is focused on the pre-cut macroscopic crack in the sample rather than on the microscopic crack precursors within the rubber.

**Figure 6:**
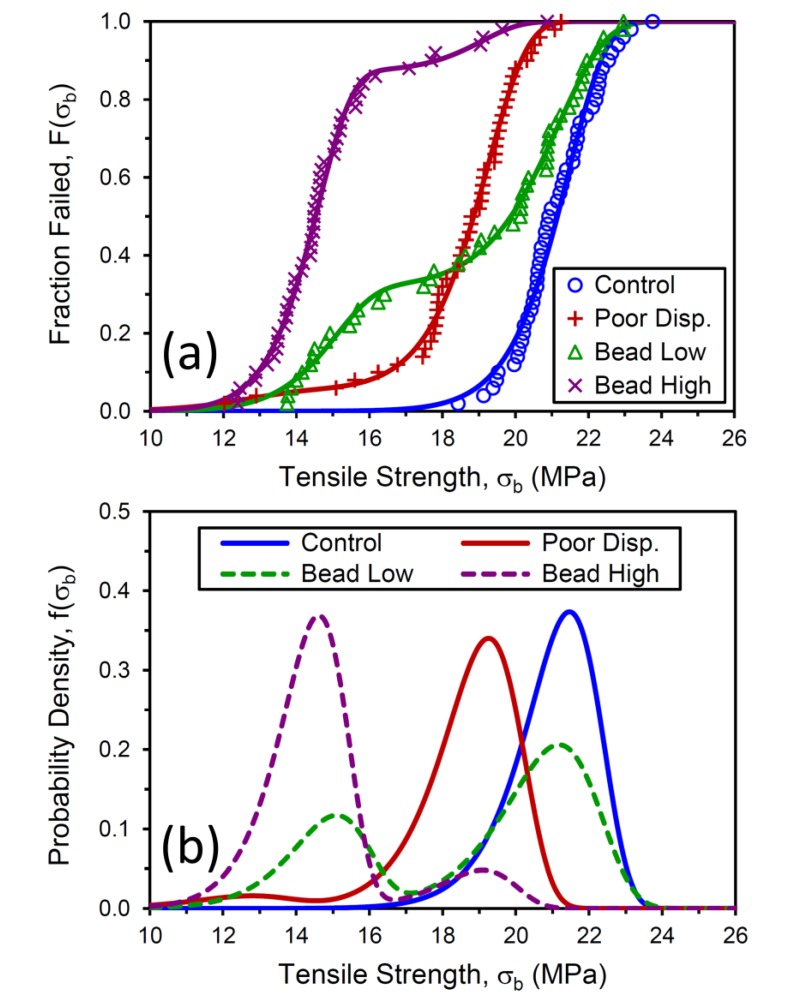
Measured tensile strength distributions for the four compounds (symbols) and Weibull cumulative distribution function fits (lines) (**a**). Associated Weibull probability density functions (**b**).

Tensile strength results from the 50 repeat tests were sorted from low to high values to produce fraction failed, *F*, versus σ_b_ responses (Figure 6a). The strength-reducing effects of poor carbon black dispersion and addition of the 0.5 mm glass inclusions are very evident in the results. The Bead Low material exhibits a bimodal σ_b_ population, which is reasonable because there was statistically less than one bead per test sample, so some specimens failed normally like the Control, and others had reduced strengths due to a large crack precursor from the presence of a glass microsphere in the gauge section of the specimen. The Bead High compound contained an average of over six glass microspheres per dumbbell gauge region, so the resulting failure distribution is fairly narrow, but shifted to much lower σ_b_ relative to the Control. The Poor Disp. compound has a σ_b_ distribution, which is generally shifted downward by about 2 MPa compared to the control, with a low σ_b_ tail that overlaps with the Bead High distribution. One important outcome from this exercise is the confirmation that the population size of 50 test specimens suggested by Wong et al. [10] is sufficient to capture the variation in tensile strength within each material for these four diverse failure distributions. There are no significant gaps between experimental data points in Figure 6a, and the results for each compound transition essentially smoothly from 0 to 1 on the fraction failed axis.

A combination of the tear testing and tensile testing results can be used to evaluate the crack precursor size distribution. The tearing energy or energy release rate, *T*, depends on the crack size, *c*, and the strain energy density, *W*:

*T* = 2 *kWc*, edge crack (1)

*T* = *kWc*, internal crack (2)

For simple tension deformation mode, the proportionality constant, *k*, is approximately related to the tensile strain (ε) by [2]:

(3) $$k\approx \frac{\pi }{{\left(1+\varepsilon \right)}^{1/2}}$$

These expressions can be used to determine the size of crack precursor, *c*_0_. The quantity k W increases as a specimen is stretched during a tensile test, where *W* is the integrated area under the stress–strain curve. When the product of this quantity and the largest crack precursor in the gauge section of the test specimen reaches the tearing limit of the material—the critical tearing energy (*T*_c_)—the sample ruptures. The breaking conditions in a tensile test, *W*_b_ and ε_b_, are thus linked to the tear strength, *T*_c_, of the rubber through *c*_0_ (combining Equations (2) and (3) for *c* = *c*_0_):

(4) $$c_{0}=\frac{T_{c}{\left(1+\varepsilon_{b}\right)}^{1/2}}{\pi \;W_{b}},\;\mathrm{internal}\;\mathrm{crack}$$

**Figure 7:**
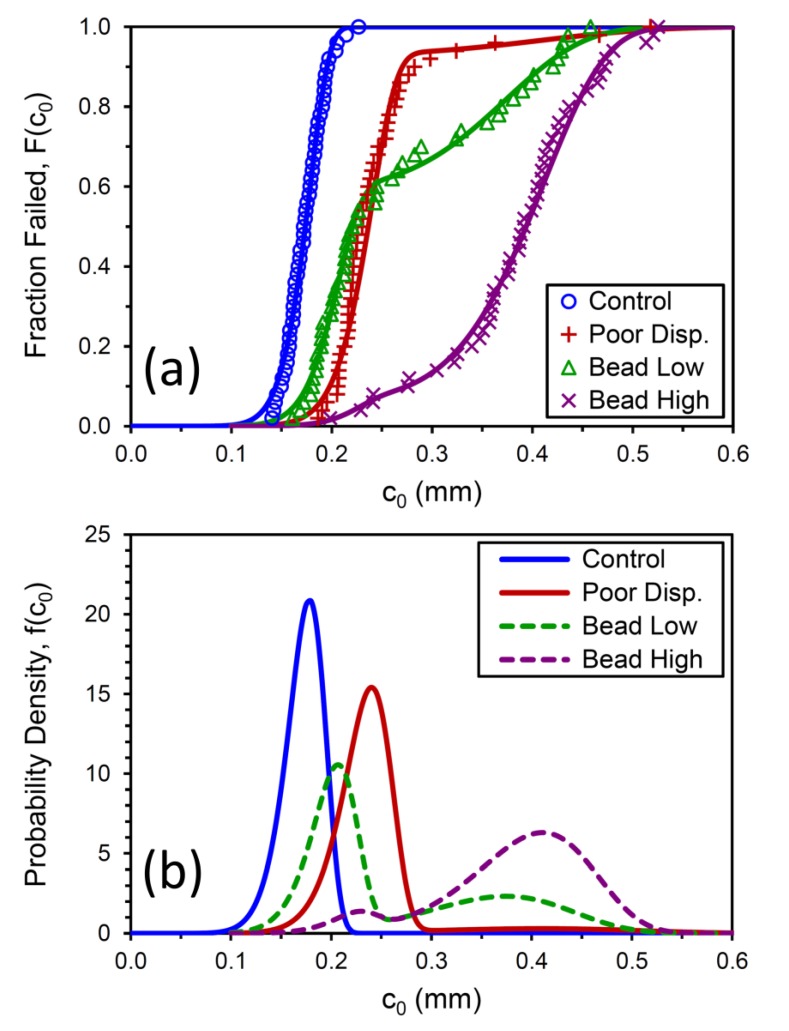
Measured crack precursor size (*c*_0_) distributions for the four compounds (symbols) and Weibull cumulative distribution function fits (lines) (**a**); associated Weibull probability density functions (**b**).

**Figure 8:**
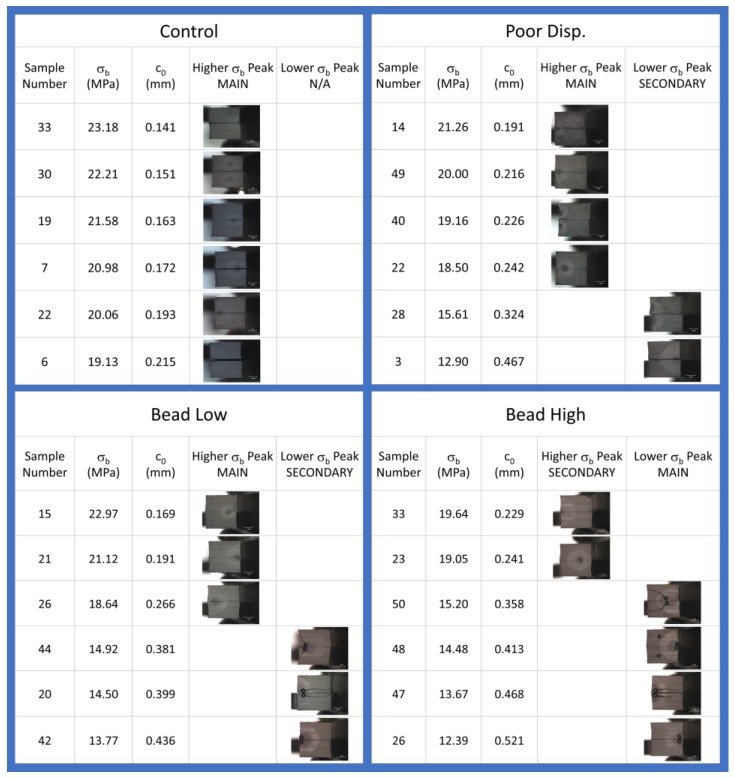
Representative fracture surfaces for tensile test specimens (two matching fracture sides shown stacked in mirror form for each) with corresponding values of σ_b_ and *c*_0_.

**Figure 9:**
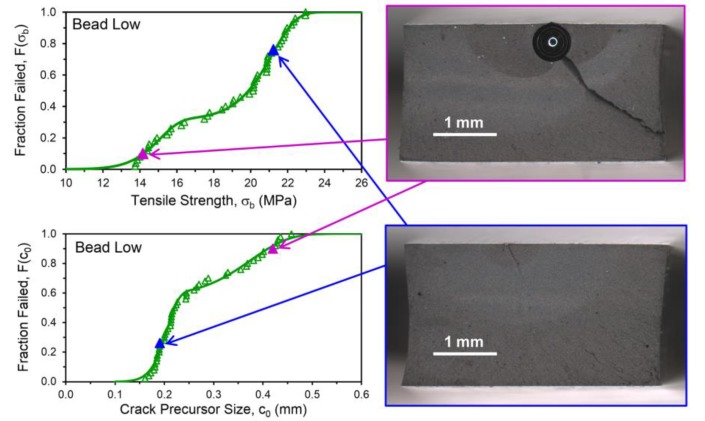
Tensile specimen fracture surfaces for two representative replicates of the Bead Low compound along with indications of their σ_b_ and *c*_0_ values in the distributions. The fracture of the specimen with the lower σ_b_ and higher *c*_0_ was clearly initiated by the presence of a glass microsphere (upper right image).

**Figure 10:**
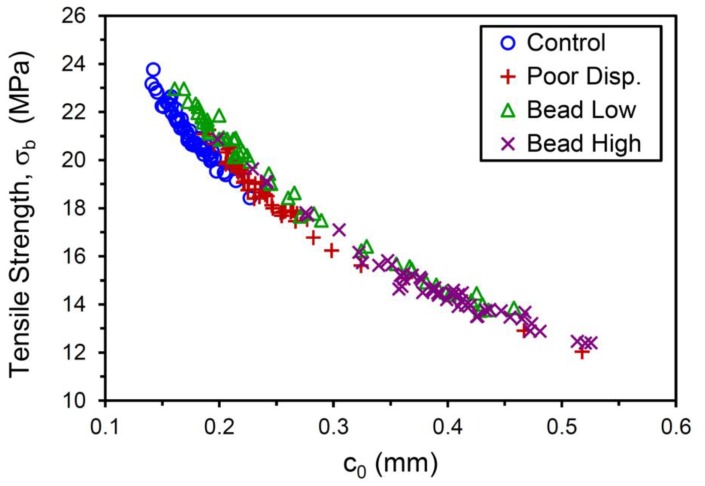
Tensile strength versus crack precursor size for the four compounds.

Values of *c*_0_ were accordingly determined for the 50 tensile test replicates for each compound, sorted from low to high, and plotted in Figure 7a. The distinctly bimodal nature of the tensile strength population noted for the Bead Low compound is also observed for the crack precursor size distribution for this material. Investigating the fracture surfaces of the tensile specimens after testing revealed that the lower values of σ_b_ and larger *c*_0_ for the Bead Low and Bead High materials were caused by the presence of the glass microspheres, and this is illustrated in Figure 8 and Figure 9. The inverse relationship between σ_b_ and *c*_0_ can be noted by comparing their cumulative distribution functions, *F*(σ_b_) and *F*(*c*_0_), which are in reverse order for the four materials (Figure 6a versus Figure 7a), and this is also pointed out explicitly in Figure 10. Lower tensile strengths come from larger precursors.

The stress at break and strain at break are commonly reported from standard tensile testing in most rubber laboratories, whereas *W*_b_ is not typically included in a results summary for a material and raw data are often not readily available for integrating. For rubber compounds with typical stress-strain curve shapes, the following estimate for *W*_b_ can be used in Equation (4) for situations where actual integrated stress–strain areas are not available:

(5) Wb≈12σb εb

**Figure 11:**
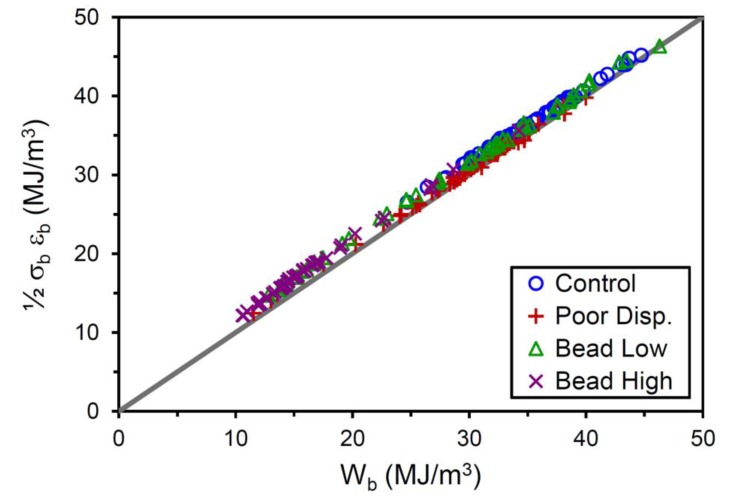
Approximation for strain energy density at break (*W*_b_ ≈ ½ σ_b_ ε_b_) versus actual *W*_b_ for the four compounds. The solid line shows equivalence of the two quantities.

This is the triangular area formed beneath a straight line drawn from the ε = 0, σ = 0 origin to the break point on the stress–strain plot. This approximation is quite good for the materials studied here, as verified in Figure 11. Incorporating this estimate for *W*_b_ in Equation (4) leads to the realization that tensile strength and tear strength are connected through crack precursor size: *c*_0_ ~ *T*_c_/σ_b_.

Weibull statistics are commonly used to represent strength and fatigue lifetime results for many classes of materials including elastomers. As mentioned earlier, this was the approach of Ignatz-Hoover and coworkers [8,9,10] for characterizing the σ_b_ distributions of rubber compounds for evaluating dispersion quality for fillers and additives. The Weibull cumulative distribution function (fraction failed, *F*(*x*)) expressions for a variable x in unimodal [14,15] and bimodal cases [16,17] are as follows:

(6) $$F\left(x\right)=1-exp\left[-{\left(\frac{x}{x_{s}}\right)}^{m}\right],\;\mathrm{unimodal}$$

(7) $$F\left(x\right)=\phi \left\{1-exp\left[-{\left(\frac{x}{x_{s,1}}\right)}^{m_{1}}\right]\right\}+\left(1-\phi \right)\left\{1-exp\left[-{\left(\frac{x}{x_{s,2}}\right)}^{m_{2}}\right]\right\},\;\mathrm{bimodal}$$

The *x*_s_ is the characteristic value or scaling constant, m is the stretching exponent, and ϕ is the fraction (from 0 to 1) that separates the two components of a bimodal population. The reliability function (fraction survived, *R*(*x*)) is simply related to *F*(*x*):

(8) R(x)=1−F(x)

The probability density function is the derivative of the cumulative distribution function:

(9) $$f\left(x\right)=\frac{dF\left(x\right)}{dx}$$

(10) $$f\left(x\right)=\frac{m}{x_{s}}{\left(\frac{x}{x_{s}}\right)}^{m-1}exp\left[-{\left(\frac{x}{x_{s}}\right)}^{m}\right],\;\mathrm{unimodal}$$

(11) $$f\left(x\right)=\phi \left\{\frac{m_{1}}{x_{s1}}{\left(\frac{x}{x_{s1}}\right)}^{m_{1}-1}exp\left[-{\left(\frac{x}{x_{s1}}\right)}^{m_{1}}\right]\right\}+\left(1-\phi \right)\left\{\frac{m_{2}}{x_{s2}}{\left(\frac{x}{x_{s2}}\right)}^{m_{2}-1}exp\left[-{\left(\frac{x}{x_{s2}}\right)}^{m_{2}}\right]\right\},\;\mathrm{bimodal}$$

The experimental data in Figure 6a and Figure 7a were fit using the Weibull *F*(*x*) function for *x* = σ_b_ and *x* = *c*_0_, and the fitting parameters are summarized in Table 3 and Table 4. The Control data were successfully captured with a unimodal Weibull fit, but bimodal distributions were necessary for the other three materials. These fits are represented by the solid lines in Figure 6a and Figure 7a. The fitting results from *F*(*x*) were then used to produce the probability density *f*(*x*) curves for each material shown in Figure 6b and Figure 7b. The reference compound exhibits a narrow precursor distribution centered around *c*_0_ = 180 μm, and adding the glass beads to the rubber produced a new population in the vicinity of 400 μm.

Conventional filler dispersion testing (Table 5) did not detect the glass beads and yielded a fairly high dispersion index of 88.3 for the Poor Disp. compound wherein 40% (20 phr) of the carbon black was added just before the last minute of the final mixing stage. The IFM dispersion method analyzes an area of 825 μm × 825 μm of a cut rubber surface, which is repeated 10 times. This gives an overall probed area of 6.8 mm^2^ in comparison to the total analyzed volume of 10,500 mm^3^ in the gauge sections of the 50 tensile specimens. Characterizing tensile strength distribution is obviously an important complementary approach to traditional filler dispersion techniques for identifying the presence of minor amounts of large inclusions in rubber.

The observation that the addition of the 517 μm diameter microspheres results in a smaller size of about 400 μm for *c*_0_ may be related to the expectation that the spherical glass beads introduce smooth, large diameter crack starters rather than sharp cracks with high stress concentrations [18]. Also, based on microscopic diagnostics of crack initiation and growth from carbon black agglomerates, Huneau et al. [4] proposed that rubber debonding first occurs at the poles of a precursor in a direction parallel to the loading, and, after additional fatigue, the crack direction eventually transitions to grow in the perpendicular direction to the load cycle input. This is an additional process that requires extra strain energy, and this may explain why the crack precursor size inferred from fatigue and strength measurements could be smaller in size than the actual physical initiator within the rubber. Other complexities may exist, including formation of nano- and micro-voids in front of a crack [19,20,21].

Characterizing the precursor distributions in the manner shown here can be valuable for exploring rubber product reliability issues using elastomer fatigue simulations with critical plane analysis [22,23,24]. A key input to such fatigue modeling is *c*_0_, and knowing its distribution allows a comparison of the predicted lifetime, for example, due to the most probable *c*_0_ versus worst case scenarios involving the large-*c*_0_ tail of the distribution. Large precursors are only an issue if they end up in the areas of the product where crack growth driving forces are highest during use. Therefore, volume concentration and spatial statistics effects could be additionally included in modeling via Monte Carlo approaches by statistically mapping a population of precursors onto the rubber part geometry.

## 4. Conclusions

The addition of 0.5 mm diameter glass beads at two levels and the impact of intentional poor mixing of carbon black were all successfully diagnosed in CB-filled SBR compounds using tensile strength testing of 50 replicate specimens. The tensile strength and precursor size distributions were captured with a unimodal Weibull function for the reference material, but bimodal functions were needed to fit the distributions for the compounds containing the microspheres and the material with poor carbon black dispersion. Critical tearing energy (tear strength) was not influenced by the presence of the CB agglomerates and glass microspheres that caused reductions in tensile strength, because the strain energy focuses on the pre-cut macroscopic crack in the sample during tear testing rather than on the microscopic crack precursors within the rubber. Despite their substantial impact on *c*_0_ and σ_b_ distributions, the glass microspheres were not detected by a conventional interferometric microscopy filler dispersion technique, which highlights the utility of the tensile strength distribution approach to identify the presence of small amounts of large inclusions in rubber.

## Figures and Tables

**Table 1:** Rubber Compound Formulations (phr).

		Control	Poor Disp.	Bead Low	Bead High
Master Batch (Non-Productive)	SBR 1500	100	100	100	100
	N550 CB (0 ppm residue)	50	30	50	50
	Zinc Oxide	2	2	2	2
	Stearic Acid	1	1	1	1
	6PPD A.O.	1	1	1	1
Final Batch (Productive)	TBBS	2	2	2	2
	Sulfur	2	2	2	2
	N550 CB (0 ppm residue)		20		
Two-Roll Mill	Glass Beads (d = 517 μm)			0.09	0.72

**Table 2:** Ultimate Tensile and Tear Properties.

		Control	Poor Disp.	Bead Low	Bead High
Tensile Failure (no initial cut)	σ_b_ (MPa)	21.13 ± 1.12	18.56 ± 1.79	18.87 ± 2.99	14.91 ± 1.85
	ε_b_	3.41 ± 0.24	3.23 ± 0.34	3.08 ± 0.54	2.39 ± 0.29
	*W*_b_ (MJ/m^3^)	34.74 ± 4.66	29.68 ± 5.73	28.23 ± 9.78	16.19 ± 4.73
Tear (initial cut)	*T*_c_ (kJ/m^2^)	8.93 ± 0.66	10.71 ± 1.01	10.43 ± 0.02	10.15 ± 0.27

**Table 3:** Weibull Fitting Parameters for σ_b_ Distributions.

	Control	Poor Disp.	Bead Low	Bead High
ϕ	1 (unimodal)	0.05306	0.3152	0.8709
σ_b,s1_ (MPa)	21.51	12.84	15.15	14.66
m_1_	21.82	10.07	14.98	16.82
σ_b,s2_ (MPa)	--	19.31	21.25	19.15
m_2_	--	18.83	17.34	19.37

**Table 4:** Weibull Fitting Parameters for *c*_0_ Distributions.

	Control	Poor Disp.	Bead Low	Bead High
ϕ	1 (unimodal)	0.9267	0.5859	0.06181
*c*_0,s1_ (mm)	0.1801	0.2424	0.2087	0.2280
m_1_	10.18	10.85	9.884	10.75
*c*_0,s2_ (mm)	--	0.4322	0.3865	0.4186
m_2_	--	4.646	5.812	7.576

**Table 5:** Interferometric Microscopy (IFM) Filler Dispersion Results *.

	Control	Poor Disp.	Bead Low	Bead High
IFM Dispersion Index [max. = 100]	99.5	88.3	99.3	99.1
Peak & Valley Area Percentage (%)	0.17	3.84	0.23	0.31
Number of Peaks & Valleys	32	1021	34	57
Mean Peak & Valley Diameter (μm)	15.0	15.2	17.4	17.2

* performed according to ASTM D2663 Method D; total scan area = 6.8 mm^2^.
